# Expression Analysis of Phenylpropanoid Pathway Genes and Metabolomic Analysis of Phenylpropanoid Compounds in Adventitious, Hairy, and Seedling Roots of Tartary Buckwheat

**DOI:** 10.3390/plants11010090

**Published:** 2021-12-28

**Authors:** Minsol Choi, Ramaraj Sathasivam, Bao Van Nguyen, Nam Il Park, Sun-Hee Woo, Sang Un Park

**Affiliations:** 1 Department of Crop Science, Chungnam National University, 99 Daehak-ro, Yuseong-gu, Daejeon 34134, Korea; 201802785@o.cnu.ac.kr (M.C.); ramarajbiotech@gmail.com (R.S.); 2 Department of Smart Agriculture Systems, Chungnam National University, 99 Daehak-ro, Yuseong-gu, Daejeon 34134, Korea; nguyenvanbao@tuaf.edu.vn; 3 Division of Plant Science, Gangneung-Wonju National University, 7 Jukheon-gil, Gangneung 25457, Korea; nipark@gwnu.ac.kr; 4 Department of Crop Science, Chungbuk National University, Cheongju 28644, Korea; shwoo@chungbuk.ac.kr

**Keywords:** buckwheat, *Fagopyrum tataricum*, *Agrobacterium rhizogenes*, auxins, phenolic compounds, root culture

## Abstract

Tartary buckwheat (*Fagopyrum tataricum*) is an important crop that belongs to the Polygonaceae family, whose roots have received considerable attention due to the presence of compounds with high nutritional and medicinal value. In this study, we aimed to develop an efficient protocol for the culture of adventitious (ARs) and hairy (HRs) roots on a half-strength Schenk and Hildebrandt (SH) medium containing different concentrations of the auxins, α-naphthaleneacetic acid (NAA), indole-3-butyric acid (IBA), and indole-3-acetic acid (IAA). The highest percentage of root induction (91.67%) was achieved with 0.5 mg/L IAA, whereas the greatest number of roots was found in 1 mg/L IAA. In contrast, 0.1 mg/L IBA returned the longest roots. As expected, HRs were obtained from in vitro leaf explants infected with *Agrobacterium rhizogenes* R1000. Quantitative real-time polymerase chain reaction (qRT-PCR) analysis of 11 phenolic pathway genes revealed that five genes (*FtPAL*, *FtC3H*, *FtHQT*, *FtCHS*, and *FtANS*) were highly expressed in HRs, whereas only four (*FtC4H*, *FtFLS2*, *FtDFR*, and *FtANR*), and three (*Ft4CL*, *FtCHI*, and *FtF3H*) were recognized in the ARs and seedling roots (SRs), respectively. HPLC analysis of phenolic compounds in different root cultures showed that the majority of the phenolic compounds (both individual and total) were significantly accumulated in the HRs. Principal component analysis (PCA) identified differences among the three root types, whereby HRs were separated from ARs and SRs based on the amount of phenolic compounds present. Analysis of the metabolic pathway revealed that among the identified metabolites, the 3, 2, and 1 pathways were associated with flavonoid, flavone and flavonol, and phenylpropanoid biosynthesis, respectively. Hierarchical clustering analysis and the heat map showed that the different root cultures presented unique metabolites.

## 1. Introduction

Buckwheat (*Fagopyrum* sp.) is an important crop belonging to the Polygonaceae family that contains compounds with abundant nutritional and medicinal value. There are two species of cultivated buckwheat: common buckwheat (*Fagopyrum esculentum* Moench.) and Tartary buckwheat (*Fagopyrum tataricum* Gaertn.). Tartary buckwheat is mainly cultivated in southern Bhutan, China, Nepal, and northern India [1], and it is richer in phenolic compounds, particularly rutin, than common buckwheat. Tartary buckwheat contains diverse compounds that are widely used in pharmaceutical applications for their anti-oxidative, anti-diabetic, anti-hypertension, anti-cancer, and cholesterol-lowering activities [2,3]. A previous study reported that the ‘Hokkai T10’ (*Fagopyrum tataricum*) cultivar can accumulate a higher amount of phenolic and anthocyanins compounds than the ‘Hokkai T8’ cultivar [2]. The production of a large quantity of valuable secondary metabolites is of great benefit in the pharmaceutical industry. Therefore, it is important to develop an efficient strategy to obtain these compounds by analyzing the components and profiling the gene expression involved in the metabolic pathway. 

Hairy roots (HRs) are caused by *Agrobacterium rhizogenes* transferring their root-inducing (Ri) plasmid into the host plant genome, including the genes capable of morphological and biological changes. Modifying the plant metabolism changes the hormonal balance, causing a proliferation of roots that emerge at the injury site [4]. Hairy root syndrome appears in many plant species exposed to *A. rhizogenes*, resulting in what is known as hairy root disease [5]. The genetic transformation does not negatively affect the synthetic ability of natural roots, and hairy roots have the ability to produce valuable secondary metabolites and grow vigorously on medium without hormones [6]. 

Adventitious roots (ARs) arise from non-root tissue, and the developmental process is affected by biotic factors for normal growth of plants as well as abiotic stress conditions, such as flooding, drought, nutrient deprivation, and wounding [7]. Adventitious roots are capable of synthesizing phytochemicals in vitro; therefore, they have high potential for use in culture systems. Plant hormones play a crucial role in adventitious root formation, particularly through the regulation of the auxin/cytokinin balance [8]. The induction of adventitious roots has been studied and developed in many species to produce important plant secondary metabolites [9]. 

Phenolic compounds are classified as chemical molecules containing at least one aromatic ring that are separated into several groups based on their structures. Some examples are phenolic acids, flavonoids, coumarins, curcuminoids, lignans, stilbenes, tannins, and quinones [10]. These molecules are widespread in the plant kingdom, primarily in the form of secondary metabolites that are involved in various physical and physiological functions. Lignins are found in the cell wall of plant tissues, providing physical strength and protection to the plant. Flavonoid anthocyanins are pigments that color plant tissues red, blue, and purple, and provide biological activities that affect the life cycle of plants, such as eliminating reactive oxygen species with their powerful antioxidant properties [11]. These important properties of phenolic compounds are considered essential elements for plant defense systems against biotic and abiotic stresses.

**Figure 1:**
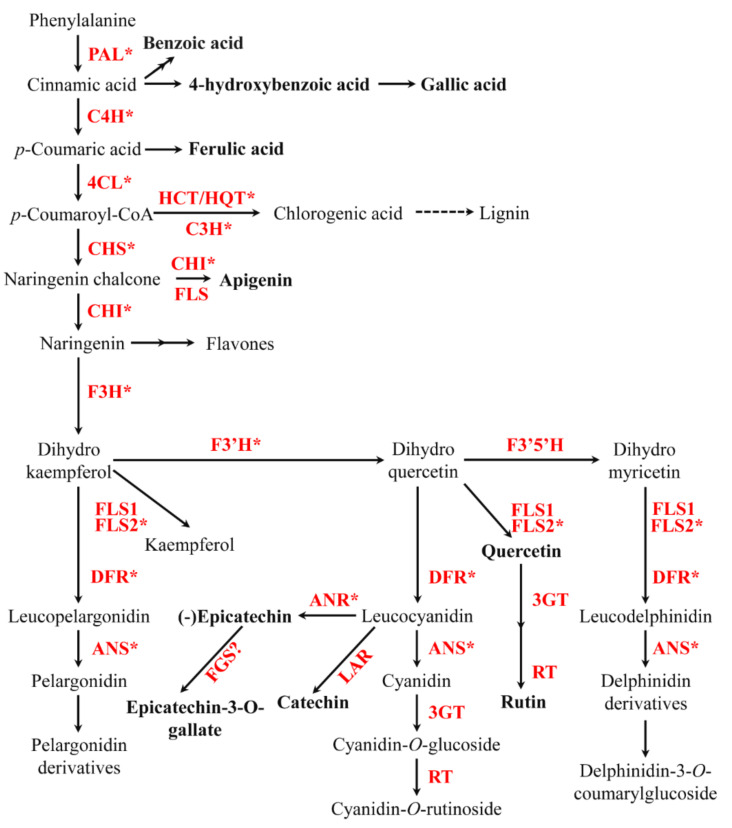
Proposed phenylpropanoid biosynthetic pathway in *F. tataricum*. The enzyme involved in each step is shown in red. The phenylpropanoid compounds measured in this study are shown in black and bold. Asterisks denote the genes used for gene expression analysis. PAL, phenylalanine ammonia-lyase; C4H, cinnamic acid 4-hydroxylase; 4CL, 4-coumaric acid: CoA ligase; HCT, cinnamoyl-CoA shikimate/quinate transferase; HQT, hydroxycinnamoyl CoA:quinate hydroxycinnamoyl transferase; C3H, p-coumaroylester 3-hydroxylase; CHS, chalcone synthase; CHI, chalcone isomerase; F3H, flavanone 3-hydroxylase; FLS, flavonol synthase; F3′H, flavonol 3′ hydroxylase; F3′5′H, flavonoid 3′,5′-hydroxylase; DFR, dihydroflavonol 4-Reductase; LAR, leucocyanidin reductase; ANR, anthocyanidin reductase; ANS, anthocyanidin synthase; FGS, flavan-3-*O*-gallate synthase; 3 GT, flavonoid-3-*O*-glucosyltransferase; RT, flavonoid 3-*O*-glucoside-6-*O*-rhamnosyltransferase. The pathway scheme is adapted and modified from Li et al. [12].

Phenolic compounds are synthesized by the phenylpropanoid pathway, which converts phenylalanine to cinnamic acid with the help of phenylalanine ammonia-lyase (PAL) [12]. Subsequently, cinnamic acid is converted to *p*-coumaric acid by hydroxylation with cinnamate 4-hydroxylase (C4H). This *p*-coumaric acid is then catalyzed by 4-coumaroyl CoA ligase (4CL) for the production of *p*-coumaroyl CoA, which is a key precursor that is helpful in the synthesis of phenylpropanoid compounds using various chemical reactions (Figure 1). Chalcone synthase (CHS) is involved in the first step of flavonoid biosynthesis. The enzyme catalyzes the formation of naringenin chalcone by condensing *p*-coumaroyl CoA with three molecules of malonyl-CoA, followed by the conversion of this naringenin chalcone into naringenin by chalcone isomerase (CHI). The enzyme flavanone 3-hydroxylase (F3H) then catalyzes the naringenin to dihydrokaempferol, which is divided into two steps for the synthesis of leucocyanidin and dihydroquercetin by the enzymes dihydroflavonol 4-reductase (DFR) and flavonoid 3′-hydroxylase (F3′H), respectively. The first step involves anthocyanidin synthase (ANS), which aids in the conversion of leucocyanidin to cyanidin, whereas the second step is linked to the flavonol synthase (FLS), which catalyzes the conversion of dihydroquercetin to quercetin. Finally, rutin is derived from quercetin by glucosyltransferase (GT) and rhamnosyl transferase (RT) [12].

To date, a few studies have reported on the metabolic analysis of *F. tataricum* roots; however, none have been identified that have compared the metabolic profiles of three types of root cultures (HRs, ARs, and SRs) using different methods. In this study, we focused on developing the optimal method for the induction of various root cultures for the production of secondary metabolites. We induced HRs, ARs, and SRs from ‘Hokkai T10′ and identified differences in their molecular and metabolomic levels. Quantitative real-time PCR (qRT-PCR) was performed to compare the expression levels of genes involved in the phenylpropanoid biosynthetic pathway. In addition, the phenolic content of each root was measured by high-performance liquid chromatography (HPLC). The result of this study could be helpful in the quest for useful secondary metabolites.

## 2. Results and Discussion

### 2.1. Optimization of Culture Medium for ARs Induction

**Figure 2:**
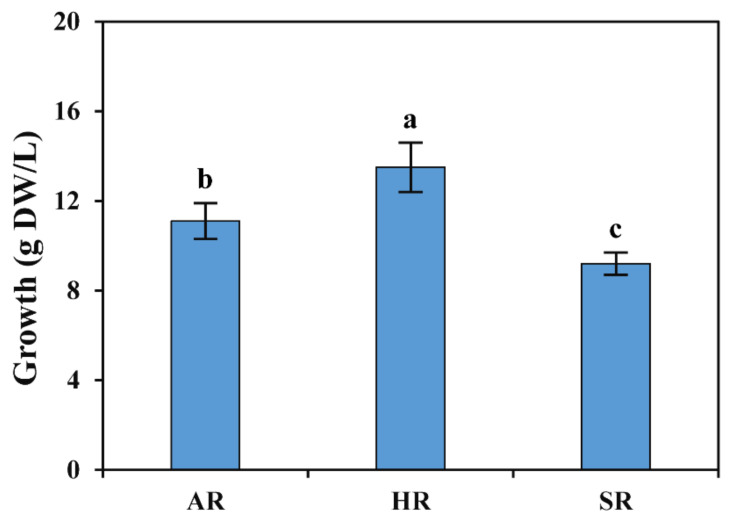
Growth of different types of root cultures of *F. tataricum* in a flask. Values followed by different letters within a column indicate a significant difference (*p* < 0.05) between areas for that parameter using DMRT (*n* ≥ 5, mean ± SD).

Auxin is a well-known phytohormone that can be beneficial when stimulating ARs [13,14]. Although there are several reports on plants in which exogenous or artificially applied auxins trigger ARs, it is necessary to test the different concentrations and select the suitable level for a species with no prior available data. Hypocotyl explants were placed in half-strength SH medium containing different concentrations of NAA, IBA, and IAA (0, 0.1, 0.5 mg/L) to determine the optimal conditions for AR induction. ARs emerged with distinctly different trends based on the auxin concentration used (Table 1). Among the hormones tested, the greatest root induction occurred with a half-strength SH medium containing 0.5 mg/L IAA (91.67%), followed by 1 mg/L IBA (83.33%), and 0.5 mg/L NAA (75%). When IBA and IAA was supplemented with half-strength SH medium, the root number and root induction percentages increased as the concentrations of IBA and IAA increased (0.1, 0.5, and 1 mg/L). A similar trend was observed in *Plumbago zeylanica*, where the root number and percentage gradually increased with incrementally-rising concentrations of auxins, such as IBA and IAA [14]. Previous studies reported that the AR number and root length were induced at minimal auxin concentrations, whereas at high levels, root induction was significantly suppressed in *Psoralea corylifolia* [15] and *Echinacea purpurea* [16]. A similar result was obtained in this study, whereby low auxin concentrations markedly increased the induction of ARs and root length. In this study, the maximum growth rates of 13.5, 11.1, and 9.2 (g dry weight (DW)/L) were achieved for HRs, ARs, and SRs grown in a flask, respectively (Figure 2). These results verify that different auxin concentrations resulted in improved AR induction. The root number was significantly higher in all the tested IAA concentrations when compared to those of NAA, IBA, and the control (half-strength SH medium). The overall result showed that a half-strength SH medium containing 0.5 mg/L IAA was suitable for AR induction in the ‘Hokkai T10′ cultivar. However, in *Boerhaavia diffusa*, the in vitro induction of ARs revealed the greatest number of roots with 1.0 mg/L NAA, whereas the most profuse rooting was observed with 2 and 4 mg/L NAA [17]. In *Robinia pseudoacacia*, the largest number of ARs was achieved with NAA, whereas in *Grewia optiva*, the IBA treatment showed superior AR induction [18]. In *P. zeylanica*, the greatest number of ARs was obtained in the medium supplemented with 1.0 and 0.5 mg/L of IBA and NAA, respectively [14]. These studies showed that a specific type of auxin might be efficient for root induction in a particular species.

HRs are otherwise called transformed root cultures, which are produced from the wound site of a stem by *A. rhizogenes* and are an excellent source for the production of high medicinal value metabolites for drug development [19]. In addition, the HRs from the mother plants show continuous vigorous growth without the application of exogenous phytohormones when compared with their non-transgenic plants [20]. A similar result was obtained in this study, that is, among the different types of root cultures the highest growth was achieved in HRs (Figure 2 and Figure S1). 

### 2.2. Phenolic Biosynthetic Pathway Gene and Its Expression Levels in Different Types of Roots

**Figure 3:**
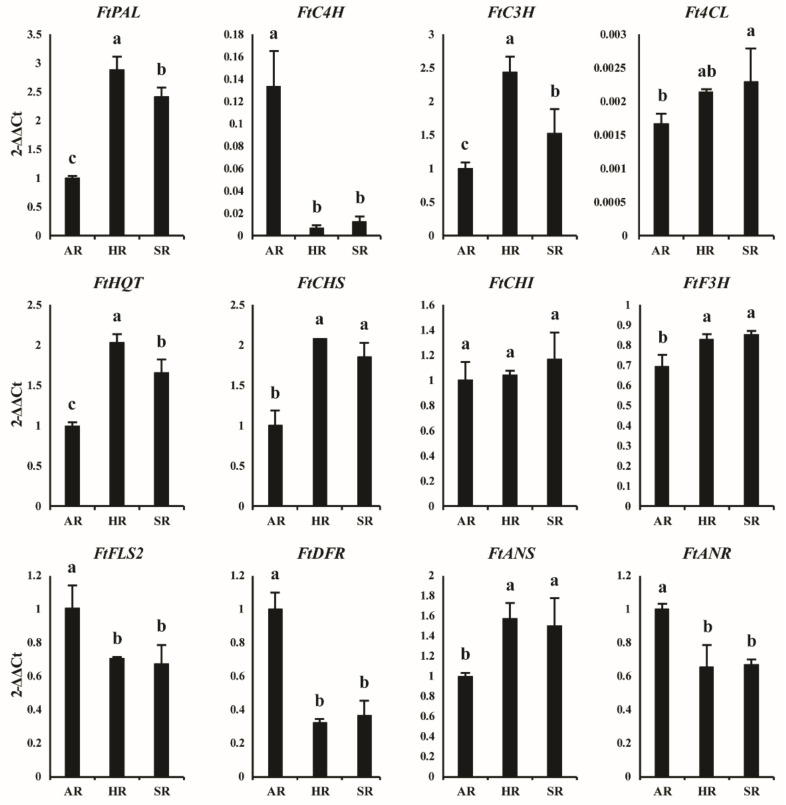
The gene expression profiles of the adventitious root (AR), hairy root (HR), and seedling root (SR) of *F. tataricum*. Values followed by different letters within a column indicate a significant difference (*p* < 0.05) between areas for that parameter using DMRT (*n* ≥ 5, mean ± SD).

We performed a qRT-PCR to determine the expression profile of phenolic biosynthetic pathway genes in the different root cultures of Tartary buckwheat. The results showed a differential expression pattern based on the specific root culture. The overall result showed that the majority of the upstream pathway genes in the HRs and the downstream genes in the ARs were highly expressed. In SR cultures, the upstream pathway genes were moderately expressed, whereas the downstream pathway genes were less expressed. Among the 11 phenolic pathway genes, the majority (*FtPAL*, *FtC3H*, *FtHQT*, *FtCHS*, and *FtANS*) were highly expressed in HRs, whereas only four (*FtC4H*, *FtFLS2*, *FtDFR*, and *FtANR*) showed higher expression in the ARs than that in SRs (Figure 3). The greatest gene expression was found in *FtPAL*, followed by *FtC3H*, *FtHQT*, and *FtCHS*, while the lowest occurred in the *Ft4CL* gene. Interestingly, all genes showed greater expression in HRs than in ARs and SRs. This result indicates that most of the upstream phenolic pathway genes were highly expressed in the HR culture system, whereas in another study, the expression levels of genes related to phenolic biosynthesis were more significantly expressed in the SRs than in the HRs and ARs, both upstream and downstream [21]. In the same study, the expression profile of genes related to astragaloside biosynthesis revealed that, in contrast, most of the genes were highly expressed in the HRs followed by SRs, and none of the genes were highly expressed in ARs [21]. The overall results suggest that the expression patterns of different pathway genes differ based on the type of root culture of the particular species.

### 2.3. Phenolic Compound Content in Three Types of Root

The analysis of three types of root culture showed the presence of 10 phenolic compounds: gallic acid, catechin, benzoic acid, 4-hydroxybenzoic acid, (-) epicatechin, epicatechin gallate, ferulic acid, rutin, quercetin, and apigenin (Table 2 and Figure S2). The greatest individual and total phenolic content were obtained with the HR culture, and in all three root cultures, the individual phenolic compound with the largest concentration was rutin, ranging from 16.133 to 21.288 µg/g DW, whereas the lowest was apigenin with 0.002 to 0.007 µg/g DW. The total phenolic levels were higher in HRs (37.770 µg/g DW), followed by SRs (32.535 µg/g DW), and finally, ARs (31.032 µg/g DW). In the HR culture, the contents of gallic acid, catechin, 4-hydroxybenzoic acid, (-) epicatechin, ferulic acid, rutin, and apigenin were greater than those in ARs and SRs, whereas, the latter two each showed only one phenolic compound that was present at a higher level, epicatechin gallate and quercetin, respectively. The total phenolic compounds in the HRs (37.770 µg/g DW) were 1.16- and 1.22-fold greater than those in the SR and AR cultures, respectively. Similarly, the rutin content in the HRs were1.13- and 1.32-fold higher than those in the SR and AR cultures, respectively. A previous study reported that HRs have the ability to accumulate the same quantity of secondary metabolic compounds as that of the mother plants; however, in most cases, they can accumulate higher quantities than the parents [22]. This finding supports our result that the highest accumulation of phenolic compounds was achieved in HRs compared to those in the SR and AR (Table 2). In the ARs, the epicatechin gallate level was slightly higher than that of the HRs and SRs. Quercetin concentrations in the SR and AR cultures were 1.07- and 1.17-fold higher than those in the HR culture. Interestingly, benzoic acid was detected only in ARs and SRs. These results showed that among the different root cultures, HRs contained the greatest individual and total phenolic content levels. This result supports the gene expression result that showed that most of the upstream pathway genes were highly expressed in the HRs when compared to ARs and SRs; this might explain the highest accumulation level of phenolic compounds in the HRs. A previous study reported that the PAL gene plays an important role in the metabolism of the phenylpropanoid pathway [23]. The first key enzyme in phenylpropanoid biosynthesis is PAL, which catalyzes the conversion of L-phenylalanine to cinnamic acid, which is the rate-limiting step in phenylpropanoid metabolism due to the link between primary and secondary metabolism [24]. Previous results and this study indicate that the increase in phenolic compounds could be due to the *PAL* gene, which is responsible for phenolic accumulation in the different types of root cultures. This supports our study result that the PAL gene is highly expressed in HRs, thus leading to a significant accumulation of phenolic compounds. 

### 2.4. Metabolic Profiling of Different Types of Root Cultures

In total, 10 metabolites were identified in the different root cultures. Among these, the amounts of the rutin and epicatechin gallate phenolic compounds were significantly higher than the other metabolites in all root types, and (-)-epicatechin had the greatest concentration in HRs. In contrast, apigenin and ferulic acid showed the lowest accumulation in all root types. Overall, the results revealed that all root types had a significant accumulation of phenolic compounds (Table 2). 

**Figure 4:**
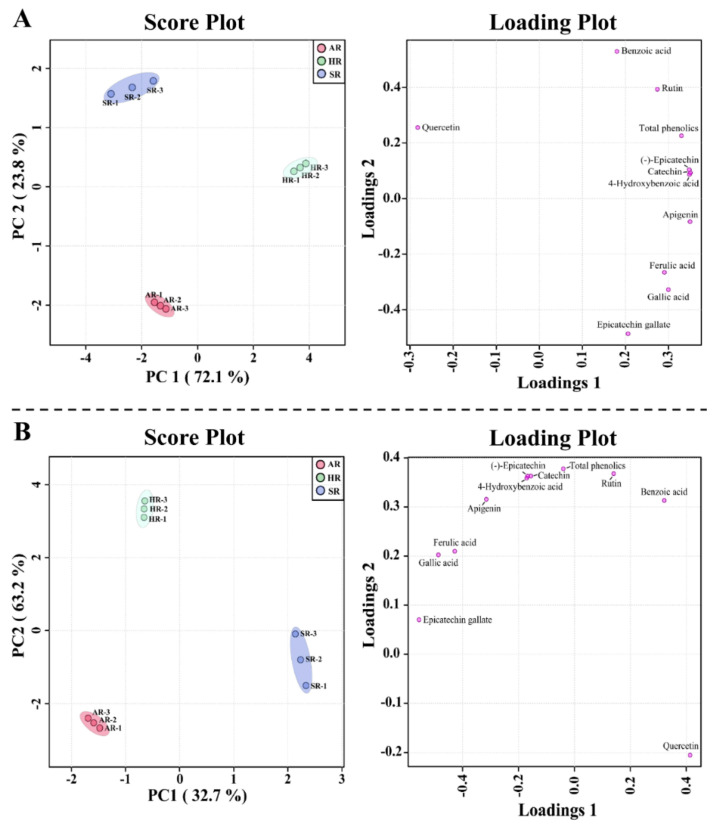
Score and loading plot of (**A**) PCA model and (**B**) PLS-DA model obtained from the metabolites detected in different root cultures of *F. tataricum*.

**Figure 5:**
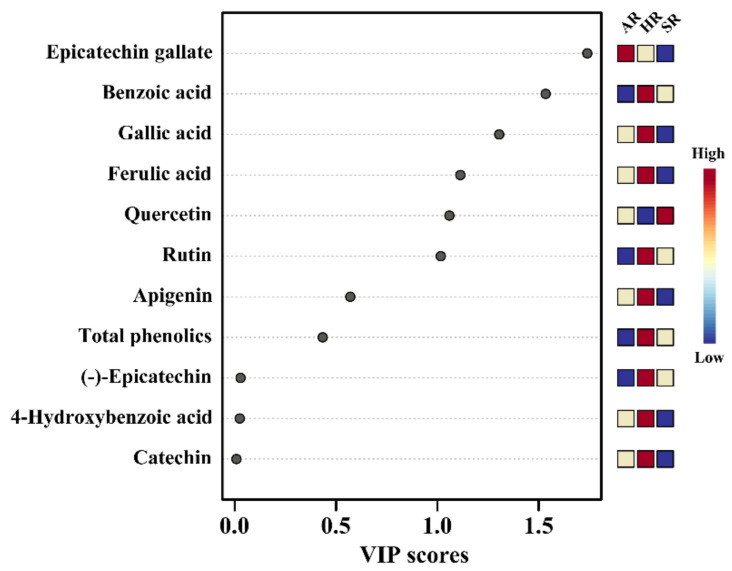
The main components separating the different types of root cultures of *F. tataricum* are based on the VIP in PLS-DA.

The above results support the PCA and PLS-DA results obtained from the root cultures, which revealed a separation of the ARs, HRs, and SRs. The PCA clearly visualized the identified metabolites that differed between the root cultures, along with the two main components (72.1% and 23.8% variance) (Figure 4A). The most important metabolite of PC1 in the root cultures was quercetin, and the associated eigenvector value was −0.28225; those of catechin, apigenin, 4-hydroxybenzoic acid, (-) epicatechin, and gallic acid were 0.35068, 0.34978, 0.34837, 0.34792, and 0.29937, respectively (Figure 4A). These metabolites determined the separation among different types of root cultures. The PLS-DA two-component analysis also showed a clear separation between the different types of root cultures of Tartary buckwheat, with 63.2% and 32.7% variance (Figure 4B). The PLS-DA results identified the most important metabolites based on the VIP value of the five-component model (Figure 5). Ten compounds were identified as discriminating metabolites in the different types of root cultures (VIP > 1). The PCA and PLS-DA results showed that the clear separation in the different types of root cultures might be due to changes in the levels of phenolic compounds.

**Figure 6:**
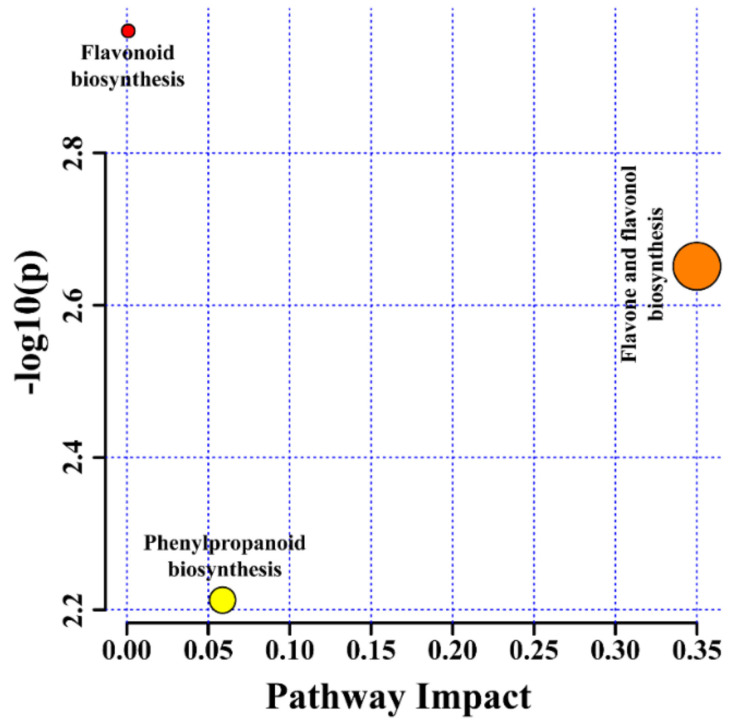
Identified metabolites and their pathway impact on different root cultures of *F. tataricum*.

Pathway impact analysis of the identified metabolites was performed using *Arabidopsis thaliana* as the source. Three pathways were identified in all the different types of root cultures (Table S1), all of which were affected (Figure 6). Among these pathways, 3, 2, and 1 were related to flavonoid, flavone and flavonol, and phenylpropanoid biosynthesis, respectively. The fact that the metabolites and their metabolic pathways were greatly impacted in the present study has nutraceutical relevance. The amino acid, carbohydrate, and fatty acid metabolism of cannabis seeds were observed to be affected in two different cultivars [25], while in another metabolomics study, organic compounds and fatty acids were mainly impacted under various irrigation regimes in chia seeds [26]. 

**Figure 7:**
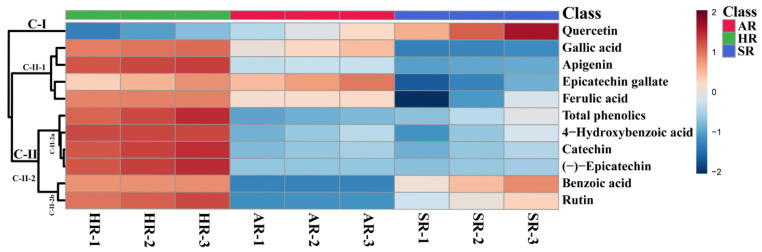
Heat map representing the change in the concentration of relative metabolites in root cultures of *F. tataricum*.

The accumulation of metabolites in different types of root cultures was elucidated based on hierarchical clustering analysis and the heat map obtained from the 10 identified metabolites (Figure 7). Two major clades were obtained with different patterns due to the greater number of altered metabolites between the different root cultures (clades I and II). Clade II was divided into two subclades (clade-II-1 and clade-II-2), of which clade II-2 was further separated into clades II-2a and II-2b. Clade I consists of quercetin, the compound present in similar amounts in all types of root cultures. Subclade CII-1 contains the compounds that were highest in the HRs and ARs. Clade-II-2a, consists of the phenolic compounds only highest in the HRs, whereas clade-II-2b comprises those highest in HRs and SRs. Clade I consists only of quercetin, whereas clade-II-1 consists of quercetin, gallic acid, apigenin, epicatechin gallate, and ferulic acid, and clade II-2 contains 4-hydroxybenzoic acid, catechin, (-)-epicatechin, benzoic acid, and rutin. The differential pattern of metabolite clustering clearly identified metabolic changes similar to the results obtained for groundnut [27], rice [28], and cannabis [25]. The significantly higher (*p* ≤ 0.05) individual metabolites in the different types of root cultures are shown in Figure S3. This result confirmed that unique metabolites are present in each of the different types of root cultures.

## 3. Materials and Methods

### 3.1. Three Types of Root Induction

Seeds of the ‘Hokkai T10′ cultivar were obtained from the National Agricultural Research Center of the Hokkaido Region. The induction of ARs and HRs originated from the hypocotyl and stem of plantlets, respectively. 

The optimal auxin concentration for inducing ARs was determined by culturing the hypocotyl explants in the dark on half-strength Schenk and Hildebrandt (SH) (150 mg/L NH_4_H_2_PO_4_, 75.5 mg/L CaCl_2_, 97.7 mg/L MgSO_4_, 1250 mg/L KNO_3_, 2.5 mg/L H_3_BO_3_, 0.05 mg/L CoCL_2_·6H_2_O, 0.1 mg/L CuSO_4_, 189.33 mg/L C_10_H_12_FeN_2_NaO_8_·3H_2_O, 5 mg/L MnSO_4_·H_2_O, 0.05 mg/L Na_2_MoO_4_·2H_2_O, 0.5 mg/L KI, 0.5 mg/L ZnSO_4_·7H_2_O, 1000 mg/L C_6_H_12_0_6_, 5 mg/L C_6_H_5_NO_2_, 0.5 mg/L C_8_H_11_NO_3_·HCl, 5 mg/L C_12_H_17_CIN_4_OS·HCl, 30 g/L sugar, 7 g/L agar, pH 5.8) containing various hormones at different concentrations. In detail, the hypocotyl explants were cut into sections of approximately 1 cm in width and height, which were placed in the half-strength SH medium with varying levels of α-naphthaleneacetic acid (NAA), indole-3-butyric acid (IBA), indole-3-acetic acid (IAA) concentrations (0, 0.1, and 0.5 mg/L). Then the cultures were maintained at 25 °C, under a 16:8-h light:dark cycle with a light intensity of 35 µmol m^−2^ s^−1^. After one month, fresh ARs were collected.

To induce the HRs, stems were infected with *A. rhizogenes* R1000, and HRs were initiated from the wounding site of the explants. Briefly, excised stems were soaked in a liquid culture of *A. rhizogenes* for 10 min, air-dried on sterile filter paper, and kept on solidified half-strength SH medium for 2 days at 25 °C in the dark. The explants were washed 6–8 times using sterile distilled water and cultivated with a half-strength SH solid medium containing cefotaxime (500 mg/L) and kanamycin (100 mg/L). After eight days, all stem explants were transferred to a half-strength SH solid medium containing 250 mg/L cefotaxime and 100 mg/L kanamycin, except for the contaminated samples. After 3 weeks, the HRs emerged from the stem explants, and were transferred to flasks containing half-strength SH liquid medium and incubated at 25 °C with continuous shaking at 110 rpm.

The surface-sterilized seeds were placed in the hormone-free half-strength solid SH medium and the cultures were maintained at 25 °C, under a 16:8-h light:dark cycle with a light intensity of 35 µmol m^−2^ s^−1^. After 3 weeks, SRs emerged from the seedlings and were collected. These three types of roots were sub-cultured in hormone-free half-strength SH liquid medium, harvested, and rinsed under running tap water. The samples were immediately stored at −80 °C All the culture experiments were carried out in triplicate.

### 3.2. qRT-PCR Analysis

Total RNA was extracted from 100 mg of ground root tissues using the Total RNA Mini Kit (Plant) (Geneaid Biotech Ltd., Taipei, Taiwan) according to the manufacturer’s instructions. Three biological replicates of the samples were collected from each root culture. One microgram of total RNA was used for cDNA synthesis with a first-strand cDNA synthesis kit (ReverTra Ace-α-, Toyobo, Osaka, Japan) following the manufacturer’s instructions. The cDNA was mixed with 2X Real-Time PCR Master Mix (BIOFACT Co., Daejeon, Korea) and run on a qRT-PCR device, CFX96 Real-Time System (Bio-Rad Laboratories, Hercules, CA, USA) for gene expression analysis. Phenylpropanoid pathway gene sequences were obtained from the transcriptomic data of *F. tataricum* in our laboratory, where DNA sequences were determined by the Illumina NextSeq500 sequencer [29]. The conditions for qRT-PCR were as follows: 95 °C for 10 min, 40 cycles with two steps: denaturation at 95 °C for 15 s, annealing and elongation at 58 °C (*FtC3H*, *FtHQT*, *FtANR*, and *FtACT*), 60 °C (*FtC4H*), 61 °C (*FtPAL*, *FtCHI*, *FtF3H*, and *FtANS*), 62 °C (*Ft4CL*, *FtCHS*, *FtDFR*, and *FtFLS2*) for 65 s, and 95 °C for 10 s; the temperature was increased by 0.5 °C per minute from 60 °C to 95 °C for detection of the melting curve. The actin was used as the internal control. The primers for analyzing the phenylpropanoid pathway genes expression were obtained from Park et al. [30] (Table S2). Each sample was evaluated in triplicate for qRT-PCR analysis. 

### 3.3. HPLC Analysis

HPLC analysis of the phenolic compounds was performed according to the protocol described by Park et al. [30]. Freeze-dried root samples were pulverized into fine powder. The extract concentration of the root samples was adjusted to 50 mg/mL with the addition of 80% HPLC grade methanol (*v*/*v*) to a 5 mL screw tube containing the powder. The tubes were vortexed and placed in a sonicator for 60 min followed by centrifugation at 16,000× *g* for 20 min at 4 °C. The crude extract was injected into a vial using a 0.45 μm syringe filter and analyzed with an HPLC instrument (NS-4000, Futecs Co., Daejeon, Korea). Phenolic compounds were separated by an Optimak column (250 × 4.6 mm, 5 µm, RStech Co., Daejeon, Korea) at 30 °C by using Agilent Technologies HPLC system (1200 series, Palo Alto, CA, USA) equipped with UV detector and the chromatogram is obtained at 280 nm. The mobile phase solvent A consists of acetic acid/methanol/water (2.5/5/92.5, *v*/*v*/*v*) and solvent B consists of acetic acid/methanol/water (2.5/2.5/95.2 *v*/*v*/*v*) with the flow rate and injection volume of 1.0 mL/min and 20 µL. The gradient program was as follows: 0% solvent B, followed by a linear gradient from 0–80% solvent B over 48 min, then holding at 0% solvent B for an additional 10 min. HPLC-grade methanol and acetic acid were purchased from Samchun Pure Chemicals Co., Pyeongtaek, Korea. All standards were purchased from Sigma-Aldrich (Sigma-Aldrich Co., St Louis, MO, USA) and purity of all standards was ≥95%. All the standards were dissolved in ethanol. For quantification and identification, a spike test was performed by adding the standard to the extracted root samples. All the HPLC analysis was done in triplicate.

### 3.4. Statistical Analysis

The mean values of the three biological replicates were statistically evaluated using the Statistical Analysis System (Version 9.4, SAS Institute, Inc., Cary, NC, USA). Significant differences among means were evaluated by analysis of variance with Duncan’s multiple range test with *p* < 0.05. Principal component analysis (PCA), partial least-squares discriminant analysis (PLS-DA), variable importance in projection (VIP), heatmap, and pathway analyses of the 10 metabolites identified in the root cultures were performed using MetaboAnalyst 5.0, with auto-scaling [31].

## 4. Conclusions

This study highlighted the production of SRs from Tartary buckwheat, ARs by exogenous application of different auxins, and HRs by *A. rhizogenes*-mediated transformation. The results showed that HR culture is highly effective in producing valuable roots in Tartary buckwheat. Although HRs were demonstrated to contain the largest amounts of phenolic compounds, ARs and SRs of Tartary buckwheat are also suitable for phenolic production, possibly using optimal conditions for yield. These methods will be highly relevant in the industrial production of useful drugs.

## Figures and Tables

**Table 1:** Effect of different types of auxins on adventitious root induction from hypocotyl explants of *F. tataricum*.

Auxin	Root Induction (%)	Number of Roots	Length of Roots (cm)
1/2 SH	66.67	5.25 ± 1.92 b	2.1 ± 0.66 ab
NAA 0.1	61.11	4.25 ± 1.09 b	1.9 ± 0.58 ab
NAA 0.5	75	2.75 ± 1.09 b	1.9 ± 0.36 ab
NAA 1	58.33	3 ± 1.22 b	2 ± 0.38 ab
IBA 0.1	66.67	4.5 ± 1.12 b	2.7 ± 1.01 a
IBA 0.5	66.67	4.75 ± 3.34 b	1.66 ± 0.58 ab
IBA 1	83.33	5.25 ± 1.48 b	1.36 ± 0.39 b
IAA 0.1	58.33	10 ± 2.74 a	2.14 ± 0.81 ab
IAA 0.5	91.67	10.25 ± 0.83 a	1.96 ± 0.68 ab
IAA 1	83.33	12.25 ± 1.48 a	1.76 ± 0.48 ab

Values followed by different letters within a column indicate a significant difference (*p* < 0.05) between areas for that parameter using DMRT (*n* ≥ 5, mean ± SD).

**Table 2:** Phenolic contents (µg/g DW) of the adventitious root (AR), hairy root (HR), and seedling root (SR) of *F. tataricum*.

Compounds	Adventitious Root (AR)	Hairy Root (HR)	Seedling Root (SR)
Gallic acid	0.699 ± 0.068 b	0.933 ± 0.019 a	0.227 ± 0.021 c
Catechin	1.616 ± 0.024 b	2.029 ± 0.026 a	1.614 ± 0.043 b
Benzoic acid	0.169 ± 0.018 a	ND	0.196 ± 0.001 a
4-hydroxybenzoic acid	0.193 ± 0.011 b	0.286 ± 0.000 a	0.192 ± 0.023 b
(-)-Epicatechin	3.922 ± 0.003 b	5.159 ± 0.087 a	3.943 ± 0.046 b
Epicatechin gallate	5.972 ± 0.109 a	5.896 ± 0.111 a	5.059 ± 0.187 b
Ferulic acid	0.042 ± 0.000 a	0.049 ± 0.000 a	0.031 ± 0.009 b
Rutin	16.133 ± 0.053 c	21.288 ± 0.314 a	18.780 ± 0.608 b
Quercetin	2.283 ± 0.053 b	2.123 ± 0.048 c	2.491 ± 0.091 a
Apigenin	0.003 ± 0.000 b	0.007 ± 0.000 a	0.002 ± 0.000 c
Total	31.032 ± 0.339 c	37.770 ± 0.605 a	32.535 ± 1.029 b

Values followed by different letters within a column indicate a significant difference (*p* < 0.05) between areas for that parameter using DMRT (*n* ≥ 5, mean ± SD).

## Data Availability

Data reported in this paper are available in the Supplementary Materials.

## Supplementary Materials

The following are available online at https://www.mdpi.com/article/10.3390/plants11010090/s1, Figure S1: Consecutive stages of hairy root (HR) induction in *Fagopyrum tataricum* ‘Hokkai T10’, Figure S2: HPLC chromatogram of standards, hairy root (HR), adventitious root, and seedling root of *Fagopyrum tataricum* ‘Hokkai T10’, Figure S3: Individual metabolites that are significantly (*p* ≤ 0.05) higher either in root cultures of *F. tataricum*, Table S1: Metabolic pathways where the identified metabolites are involved in root cultures of *F. tataricum*, Table S2: List of primers used in the qRT-PCR analysis.
