# Supplementary material for: Expression Analysis of Phenylpropanoid Pathway Genes and Metabolomic Analysis of Phenylpropanoid Compounds in Adventitious, Hairy, and Seedling Roots of Tartary Buckwheat
Source: Plants (Basel). 2021 Dec 28;11(1):90. doi: 10.3390/plants11010090 (PMC8747410; doi:10.3390/plants11010090)
Supplement: Supplementary file 1 [file plants-11-00090-s001.zip › plants-1457616-supplementary/Supplementary files/Supplementary Figures S1&S2.pdf]

Figure S1

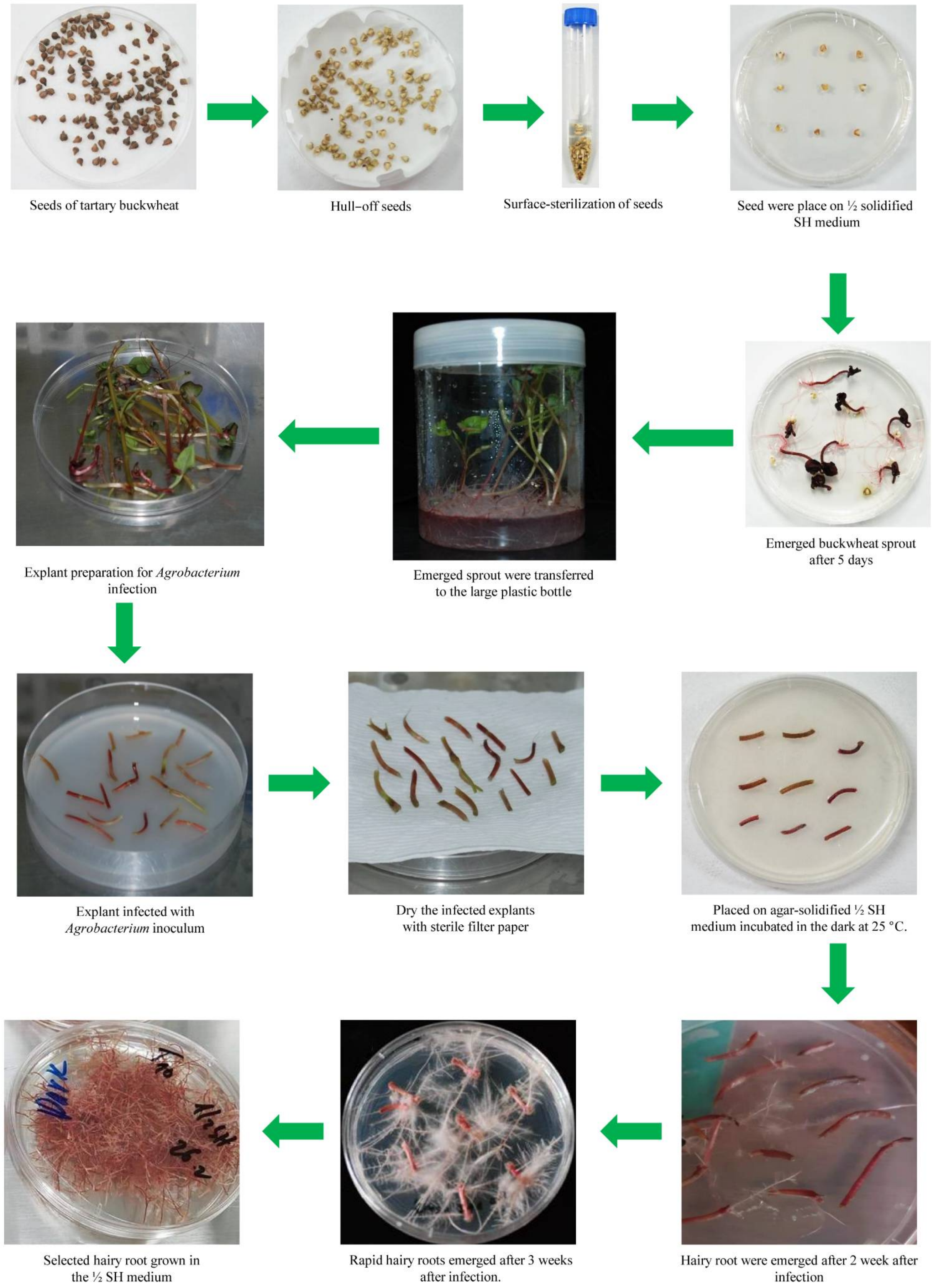

Figure S1. Consecutive stages of hairy root (HR) induction in *Fagopyrum tataricum* 'Hokkai T10'..

Figure S2

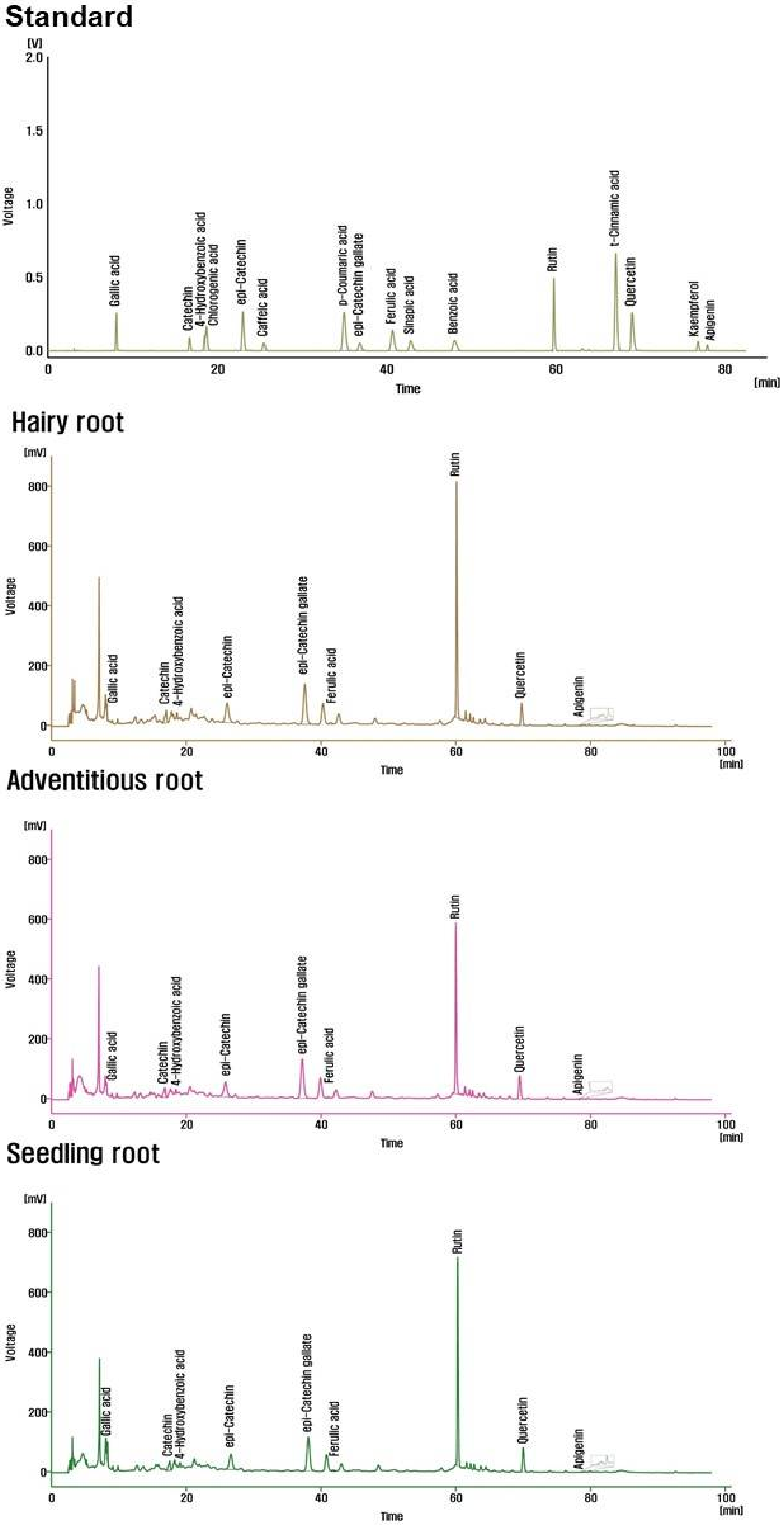

Figure S2. HPLC chromatogram of standards, hairy root (HR), adventitious root, and seedling root of *Fagopyrum tataricum* ‘Hokkai T10’..
